# Equity-oriented food supports: Learnings from the Nova Scotia COVID-19 pandemic response

**DOI:** 10.17269/s41997-024-00929-y

**Published:** 2024-09-09

**Authors:** Valerie Blair, Eleanor Eville, Christine Johnson, Heather Monahan

**Affiliations:** 1 Nova Scotia Health, Public Health, Dartmouth, NS Canada; 2 Nova Scotia Health, Public Health, Amherst, NS Canada; 3 Nova Scotia Health, Public Health, Antigonish, NS Canada

**Keywords:** Food security, Food insecurity, COVID-19 pandemic, Charitable food assistance, Emergency planning, Health inequities, Sécurité alimentaire, insécurité alimentaire, pandémie de COVID-19, aide alimentaire caritative, planification en cas d’urgence, inéquités sanitaires

## Abstract

**Setting:**

Public health measures enacted during the COVID-19 pandemic significantly impacted Nova Scotians experiencing food insecurity. Public Health (PH), Nova Scotia Health, created a provincial Housing Isolation Program (HIP) which addressed barriers to isolation, including food access, for COVID-19 cases and contacts being followed by PH.

**Intervention:**

HIP worked with partners to coordinate and respond to urgent food needs of isolating clients by providing grocery and meal delivery options. HIP also made referrals to government and community partners for income and food supports. This program was intended to minimize the spread of COVID-19 by facilitating isolation while meeting basic needs for people with no other means of support.

**Outcomes:**

From December 2020 to March 2022, HIP completed grocery and meal deliveries for 579 clients, 1351 referrals to a provincial Income Support Program, and 231 referrals to external food supports. HIP staff worked with clients to manage potential perceptions of stigma. Challenges reported included the urgency of food needs, lack of social supports, and availability and accessibility constraints in rural communities, as well as difficulty accessing culturally appropriate foods and special diets.

**Implications:**

This intervention demonstrates the importance of addressing food insecurity during emergency preparedness, planning, and response. During emergencies, planning and mobilizing food access requires an equity-oriented approach to overcome stigma. Broadly, continued reliance on charitable responses creates significant vulnerability during emergencies and addressing root causes of food insecurity through social policy will provide longer-term protection.

## Résumé

**Contexte:**

Les mesures de santé publique adoptées pendant la pandémie de COVID-19 ont eu un important impact sur la population néo-écossaise en situation d’insécurité alimentaire. Les Services de santé publique de Santé Nouvelle-Écosse ont créé un programme provincial d’hébergement en isolement (Housing Isolation Program - HIP) qui s’est attaqué aux obstacles à l’isolement, y compris l’accès à la nourriture, pour les cas de COVID-19 et les contacts suivis par la Santé publique.

**Intervention:**

Le programme d’hébergement en isolement (HIP) a travaillé avec des partenaires pour assurer la coordination et répondre aux besoins alimentaires urgents de la clientèle isolée en proposant des solutions de livraison d’épicerie et de repas. Le HIP a également orienté la clientèle vers des partenaires gouvernementaux et communautaires pour qu’elle bénéficie d’un soutien au revenu et d’une aide alimentaire. Ce programme visait à minimiser la propagation de la COVID-19 en rendant l’isolement plus facile tout en répondant aux besoins fondamentaux des personnes qui n’ont pas d’autre source de soutien.

**Résultats:**

De décembre 2020 à mars 2022, le HIP a effectué des livraisons d’épicerie et de repas pour 579 personnes; 1 351 aiguillages vers un programme provincial de soutien au revenu; et 231 aiguillages vers des soutiens alimentaires externes. Le personnel du HIP a travaillé avec la clientèle pour gérer les perceptions potentielles de stigmatisation. Parmi les difficultés signalées, citons l’urgence des besoins alimentaires, le manque de soutien social, les contraintes de disponibilité et d’accessibilité dans les communautés rurales, ainsi que la difficulté d’accès à des aliments culturellement adaptés et à des régimes alimentaires particuliers.

**Implications:**

Cette intervention démontre l’importance de la prise en compte de l’insécurité alimentaire lors de la préparation, de la planification et de l’intervention en cas d’urgence. Dans les situations d’urgence, la planification et la mobilisation de l’accès à la nourriture nécessitent une approche axée sur l’équité afin de surmonter la stigmatisation. D’une manière générale, le fait de continuer à s’appuyer sur des réponses d’ordre caritatif crée une vulnérabilité importante dans les situations d’urgence. S’attaquer aux causes profondes de l’insécurité alimentaire au moyen d’une politique sociale permettra d’assurer une protection à plus long terme.

## Introduction

At the onset of the pandemic, populations experiencing social inequities were known to have higher rates of chronic disease, placing them at greater risk for severe illness and higher mortality due to COVID-19 (Bambra et al., 2020). International and Canadian reports later confirmed that the pandemic had inequitable population health impacts (Berchet et al., 2023; Public Health Agency of Canada, 2020, 2022).

Public Health (PH) in Nova Scotia (NS) recognized that some COVID-19 measures would disproportionately impact populations experiencing social inequities such as food insecurity, precarious employment, and inadequate housing and that these impacts needed to be addressed. Our response was guided by the NS PH standards and protocols, which outline the role of PH in addressing the structural conditions and societal factors that perpetuate inequities and in mitigating their impacts on health (Government of Nova Scotia, 2011, n.d).

Inequities in health outcomes are prevalent in NS, including income-related household food insecurity (Nova Scotia, 2015). Prior to the COVID-19 pandemic, NS consistently reported higher rates of food insecurity (FI) than the national average (Tarasuk et al., 2022). Public health measures enacted in response to the pandemic such as isolation, school and business closures, and lockdowns negatively impacted people experiencing inequities (Idzerda et al., 2022).

In Canada, governments are required to have emergency plans; however, those that exist fail to robustly address food system disruptions (MacRae, n.d). To our knowledge, existing plans do not fully tackle households experiencing food access challenges such as inadequate income, distance from food sources, isolation requirements, reduced social contacts, and ability to order food online.

We are sharing how our provincial COVID response and Housing Isolation Program (HIP) observed and worked to address the isolation challenges faced by people experiencing FI, the innovations in our approach, and the need for social policies to eliminate FI. Our experiences also demonstrated that emergency planning must include equitable food access rooted in local community context and be appropriately resourced and not reliant on charitable responses. This paper describes how the emergency food needs of people isolating due to COVID-19 in NS illuminated the extent and impacts of social inequities and material deprivation during the pandemic (December 2020–March 2022).

## Setting

HIP was an equity-oriented PH pandemic response focused on creating conditions to enable successful isolation across NS. While universal public health measures like isolation and social distancing can interrupt transmission chains, they can worsen existing health inequities, whereby some people have the capacity to follow the measures, while others do not due to inequities such as food insecurity, overcrowded living conditions, limited social supports, and working conditions. Given that isolation was a directive of PH, we had a responsibility to mitigate the inequitable impacts of this measure. HIP used a proportionate universalism approach whereby each person/family was provided with the specific supports they needed to fulfill isolation directives and reduce the risk of exposures and transmission (National Collaborating Centre for Determinants of Health, 2013).

HIP team members were Nova Scotia Health (NSH) PH practitioners redeployed to the COVID-19 response. The team was experienced in health promotion strategies, understood health and social inequities, and held relationships with partners and local communities.

**Fig. 1:**
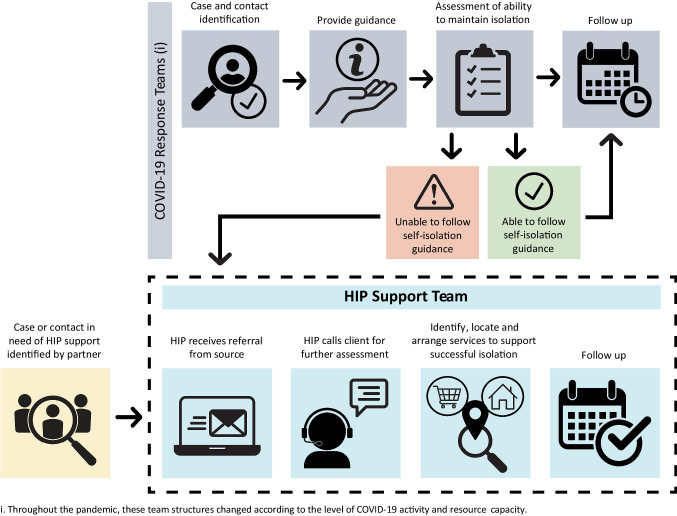
Pathway of COVID-19 assessment, referral, and response

HIP offered two streams of isolation support: (1) alternative accommodations in hotels/motels and (2) resources for people to isolate at home. NS participated in the federally funded PHAC Safe and Voluntary Isolation Spaces (SVIS) program which aimed to reduce community transmission of COVID-19 by supporting those unable to safely self-isolate (Government of Canada, 2023). There were two referral pathways to HIP: (1) externally, through community-based organizations who identified people requiring supports, and (2) internally, through the PH COVID-19 Response Teams (CRT) during initial case and contact tracing, assessment, and follow-up (Fig. 1). CRT used an assessment tool to identify the isolation supports required and shared the results with the HIP team. HIP staff would then call the person to understand their specific needs. For people whose spoken language was not English, we used language interpretation services.

As a new PH program developed during an emergency, ensuring quality improvement (QI), establishing processes, and expanding partnerships were priorities. The HIP team constantly learned, innovated, and adapted in response to the changing pandemic environment. Along with partners, we identified ways to broaden the available social supports. As we learned from clients, other PH teams, and community partners, we adjusted our processes to better meet clients’ needs. The spread of the virus across NS and into small communities provided new information and presented new challenges. Ongoing changes were documented in 38 versions of our process guidelines by the end of the program. This speaks to our commitment to QI but also to the challenge of food access across this small province. To facilitate this work, HIP created its own data collection processes and tracking database.

The key relationships that offered feedback, community-specific knowledge, and support in building new processes included:

- PH COVID-19 teams and other NSH programs
- Provincial government departments, such as NS Department of Health and Wellness (DHW) and NS Department of Community Services (DCS)
- Organizations supporting people facing health and social inequities such as Confederacy of Mainland Mi’kmaq, Mi’kmaw Friendship Centre, Immigrant Settlement Association of NS, YMCA, Association of Black Social Workers (ABSW), and the Health Association of African Canadians (HAAC)
- Service providers such as hotels, businesses, and delivery partners

## Intervention

### Addressing food needs during isolation

Initially, HIP did not include food support for people isolating at home, but it was identified as a necessity for many households during the case and contact tracing process. Charitable food responses were unable to fully meet isolation needs. As a result, NS PH allocated provincial COVID response funding to support food provisioning during isolation, which was rooted in public health values and included the principles of choice, acceptability, cultural appropriateness, and food for distinct health and social needs as key components of food security. Table 1 outlines the food and income–related supports offered through HIP.

**Table 1:** HIP’s food and income support from December 2020 to March 2022

Type of support	Description of HIP role
Referrals	• Sole referral source to the NS DCS Income Support Program (ISP), designed to help people impacted financially by isolation requirements who did not qualify for other financial support • Referral source to the DCS/Family Resource Centre Family Food Program, established in April 2021. This provided food and other household essentials for isolating families and individuals within Halifax Regional Municipality and was expanded province-wide in January 2022
Direct provision of necessities	• Provided direct food support to meet urgent needs across NS • Used online grocery delivery platforms where available, and/or grocery store pick-ups using taxis or other delivery services • Ordered prepared meals for individuals and families who could not cook or who did not have enough food at home while awaiting food delivery • Provided household essentials, such as personal hygiene products, diapers, cleaning supplies, and personal protective equipment
Navigation	• Connected people to existing food and income supports within community, such as 211, applications to federal programs like Canada Emergency Response Benefit, and a provincial sick-time coverage program

*DCS *Department of Community Services

To the best of our knowledge, the HIP approach was unique. We were the only jurisdiction in Canada providing food directly to people isolating at home. Although supporting home isolation was not in the purview of the SVIS program, we scanned other SVIS-funded programs in seven Canadian provinces and territories to learn if any had initiated home isolation supports as a complementary program to SVIS. We found that none reported supporting home isolation. We reached out to PH dietitians across Canada and learned that their roles (*n* = 3) focused on working with community coalitions and partners engaged in emergency food responses for the general community experiencing FI. A scoping review by the National Collaborating Centre for Methods and Tools (2023) confirmed the individuality and innovation of the HIP approach. Their scan of 23 initiatives in OECD countries found only one similar initiative providing direct food supports for those isolating at home in order to reduce exposure and transmission (Malmusi et al., 2022). Of the 12 initiatives aiming to address pandemic impacts included in the review, food access and food security were the most common areas of focus. However, most food-focused initiatives were described as community-based, emergency food programming for people experiencing FI generally and not direct food supports for those isolating.

## Outcomes

The population of NS is under one million, with 41.1% of people living rurally (Statistics Canada, 2022). Table 2 situates HIP supports within the broader context of the NS population and overall COVID-19 activity. From December 2020 to March 2022, HIP supported 1846 people (COVID-19 cases and contacts). While this number is relatively small, their isolation needs were complex and multifaceted and required extensive resources. People’s needs were pronounced and there were no existing emergency plans in place to adequately guide a response. Isolation requirements changed with the various waves of the pandemic (Bank of Canada, 2022). This affected PH’s ability to maintain consistent processes and shifted the volume of work and populations of focus for HIP. Our ability to track comparable data over time was limited.

**Table 2:** Profile of population required to isolate

Population of NS — July 1, 2021	992,055^i^
Estimated # COVID-19 cases required to isolate — Dec 1, 2020–March 31, 2022*	52,782^ii, iii^
Estimated # COVID-19 contacts required to isolate — Dec 1, 2020–Dec 31, 2021**	20,211^ii, iii^
# of clients (cases and contacts) supported by HIP — Dec 1, 2020–March 31, 2022	1846

*Data sourced from Panorama^iii^ (Dec 2020–2021), and provincial lab-confirmed cases (Jan 2022–March 2022)

**Data sourced from Panorama. Includes contacts that did not become cases

^i^Nova Scotia Annual Population Estimates, Nova Scotia Department of Finance and Statistics

^ii^Upon arrival of the Omicron variant in December 2021, PH could no longer create case investigations for all COVID-19 cases and as such, lab-confirmed cases became our best estimate. This does not reflect people reporting positive rapid antigen tests who would have been required to isolate and may have benefitted from HIP support. PH also stopped following contacts at this time

^iii^Panorama is Nova Scotia’s communicable disease case management and surveillance system

FI emerged as a barrier to complying with isolation measures and demonstrated how social inequities created health and socio-economic impacts for some groups of people and posed a risk to protecting the health of the general population. Characteristics of households requiring HIP support included people who were living on low/inadequate income, low-wage workers without sick-time benefits, living in crowded housing and multigenerational households, newcomers, and living in rural areas lacking transportation and reliable internet access. Households requiring HIP food support were often experiencing the most severe forms of food insecurity characterized by food access problems and reduced food intake (Government of Canada, 2020).

### Program evaluation

The HIP evaluation included a document review of ongoing HIP team written reflection activities, an online survey of HIP and CRT staff (*N* = 31; 67% response rate), and interviews with PH COVID-19 response leadership (*N* = 5). The main findings of this evaluation showed that:

- Clients were satisfied with the scope and manner in which supports were provided.
- Financial and logistical burdens of obtaining food were reduced.
- Clients expressed relief at receiving food to assist with isolation.
- The program was beneficial and contributed to improved well-being during isolation.

Formal evaluation with HIP clients isolating at home was not conducted due to logistical challenges and resource constraints. However, evaluation findings capture the observations of HIP and CRT staff who spoke regularly with those in isolation.

### Extent of food needs and food insecurity experiences

Table 3 shows HIP data from December 2020 to March 2022, including the number and type of food supports provided. HIP supported 752 people with hotel isolation and an additional 1094 people to isolate at home. Overall, 579 people were provided with food/meal/grocery deliveries. The table highlights referrals to the DCS Income Support Program for isolating individuals, as lack of income was a barrier to addressing food needs.

**Table 3:** HIP supports and referrals from December 1, 2020, to March 31, 2022

Total clients supported in isolation	1846
Clients isolating in hotel (meals included)	752
Clients isolating at home	1094
Clients receiving food/meals/groceries	579^i^
Clients referred to other food programs providing direct support (Feed NS^ii^, DCS Family Food Program)	231
DCS Income Support Program referrals	1351

^i^Clients isolating at home or in hotels where food service was not available on-site or culturally appropriate. Some clients required multiple deliveries

^ii^Feed NS is an umbrella organization for member food banks across the province. They offered a COVID-19 food box program, which offered home delivery of non-perishable food items

**Fig. 2:**
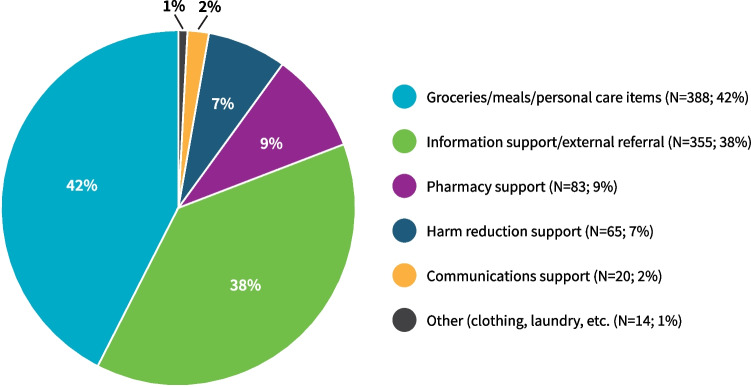
HIP supports provided to clients isolating at home by type between December 2020 and March 2022

Figure 2 shows the types of support HIP provided to people isolating at home. Food was the most frequent need (42%).

The extent of food needs reflected both material and social deprivation, exacerbated by a pandemic response that impacted income and discouraged social contacts. Experiences of FI were often due to insufficient financial resources. Households struggled with lost income, inadequate income, and limited access to food and necessities. These challenges were compounded by the shock of being identified as a case or contact and the stress of illness due to COVID-19. Difficulties accessing credit and social supports were also barriers to meeting food needs.

Food needs were urgent and ongoing throughout the isolation period. Often, people did not have enough food at home while waiting for external income and food support. HIP staff would fill these gaps to prevent missed meals. Extension of isolation due to transmission within the household required additional food deliveries.

HIP staff commonly heard that clients did not have or could not enlist help from family or friends to purchase or deliver food for them. This stemmed from a variety of factors: (1) they did not have anyone to help them; (2) they could not or did not want to financially burden anyone; (3) they did not want to disclose that they were isolating; and (4) everyone they knew were in isolation.

The shame, stigma, and stress associated with FI, as described by Williams et al. (2012), were evident. During calls, people would hesitate to ask for items they needed as they did not want to ask for too much. They expressed worry about being denied requests, asking questions such as, “Are we allowed to order popsicles for our kids?” Caregivers often prioritized their children’s food needs above their own which aligns with literature showing that caregivers will feed their children first and forgo food for themselves (Williams et al., 2012). HIP staff reassured caregivers that they also deserved to have their food needs met.

### Implementing an equity-oriented approach

Our direct approach to food support was person-centred, free of judgement, and focused on filling gaps to meet the unique needs of households in isolation. For example, HIP provided foods appropriate for varying cultural preferences, special diets, COVID-19 recovery, and those related to celebrations. HIP provided other basic needs typically unavailable through charitable food programs, such as personal hygiene products, cleaning supplies, personal protective equipment, diapering supplies, and infant formula. Our equity-oriented approaches rooted in dignity, choice, and the centring of people’s needs and preferences were later modeled by the DCS Family Food Program.

### Encountering food system challenges

While striving to meet food needs in community, HIP staff encountered many food system challenges related to physical access to fresh, affordable food. Our experience confirmed that reliable food access was a challenge in rural communities and that access to culturally appropriate foods and those for specific dietary requirements was difficult in most areas (Activating Change Together for Community Food Security, 2014). Meeting food needs in rural communities was also limited by the reality that stores with online ordering systems and delivery are not readily available in all areas. Canada has been slower to adopt online ordering platforms as a mode of accessing food resources (Mah et al., 2022). In times of heightened COVID-19 activity, demands for online ordering increased because the general population began to use these services to reduce their social contacts.

In addition, the pandemic exacerbated existing limitations of charitable food approaches, such as their volunteer and donor reliant models, their inability to meet unique food needs, and the association with stigma.

### Possible protective factors in the community

Social support systems, including community-based organizations, have a protective role in decreasing the impacts of FI. They contribute to community self-reliance by connecting community members with food, each other, and social supports (Activating Change Together for Community Food Security, 2014). Similar to previous emergency situations in NS, we witnessed families and friends helping each other during the pandemic. Trusted community organizations were a vital resource to help disseminate COVID-related information and provide isolation supports in order to keep their communities protected. These efforts likely reduced the need for HIP support. A notable example was the work of the ABSW and HAAC who quickly recognized the negative impacts that COVID-19 would present to Black communities due to ongoing systemic inequities, mistrust of the health system, and anti-Black racism. The ABSW/HAAC partnership provided culturally specific support for Black communities across the province. They established a dedicated province-wide toll-free number that people could call to ask questions and seek resources including, but not limited to, the delivery of food boxes and transportation to grocery stores. Although not an exhaustive list, other known community-based organizations that helped with isolation needs including food access were the Mi’kmaw Native Friendship Centre, Mi’kmaw Community Health Centres, Family Resource Centres, Immigrant Associations, and faith-based groups.

## Implications

The HIP experience of providing isolation support to those experiencing inequities revealed a clear picture of the pervasive and profound impact of severe food insecurity on population health. Economic turmoil during the pandemic, including inflationary pressures on food prices, affected income precarity and resulting FI, particularly for groups who were already experiencing vulnerability (Mah et al., 2022). The depth and extent of FI posed a significant challenge to following pandemic isolation measures and conversely, isolation and other associated PH pandemic measures exacerbated FI. While still early to fully assess the impacts of the pandemic on FI rates, a systematic review by Idzerda et al. (2022) concluded that FI rates increased slightly in Canadian households during the pandemic and that those most likely to be affected were households who lost employment or were job insecure and households with children. About 42% of households who had to rely on pandemic-related benefits were food insecure (Tarasuk et al., 2022).

HIP experiences showcased that FI needs to be robustly considered in public health emergency planning, preparedness, response, and recovery, recognizing that reliance on charitable responses is insufficient. The depth of material and social deprivation and access challenges demonstrates that charitable food responses were not adequate to fulfill the needs that arose during the pandemic and created vulnerability for virus transmission. Research prior to and during the pandemic showed that most food insecure households do not use food banks regularly and that FI remains severe and persistent for those who receive charitable food assistance (Men & Tarasuk, 2021). Despite this extensive evidence that charitable food responses do not address FI, governments bolstered charitable food programs by distributing $330 million dollars federally and $2.3 million provincially from the start of the pandemic to the end of 2021 (Agriculture and Agri-Food Canada, 2021; Government of Nova Scotia, 2020, 2021). Both levels of government established income support measures; however, these were rarely tied to the goal of reducing FI.

HIP has valuable learnings to inform emergency preparedness as it relates to food planning and beyond. To create effective emergency preparedness plans that include food provisioning, the HIP experience has shown that the approach needs to be multi-pronged, uniquely situated to diverse community contexts and geographies, and adequately resourced. Specifically, our approach to coordinating provincially while acting locally through partnerships and collaborations with businesses, community organizations, and government departments was a key facilitator. Further, to effectively support food needs during an emergency, the application of equity-oriented approaches rooted in dignity, choice, and the centring of people’s needs and preferences is imperative and aligns well with the “Public Health Ethics Framework: A Guide for use in Response to the COVID-19 Pandemic in Canada” (Government of Canada, 2022).

In planning for future emergency responses, public health practitioners, government agencies, and community organizations will need to work together to implement evidence-based policies and interventions that address the root causes of FI. The need to focus on income support measures, social inclusion, and robust food system infrastructure as solutions to FI is well documented (Persaud et al., 2021; Men & Tarasuk, 2021; Public Health Agency of Canada, 2020). Reducing FI and building social equity would improve population health over the long term and provide more significant health protection in the event of a pandemic or other public health emergencies.

## Implications for policy and practice

What are the innovations in this policy or program?

- Public Health filled a system gap and reduced the impact of PH measures on those experiencing food insecurity by providing direct food support and household essentials to clients with urgent needs isolating at home.
- HIP engaged in an equity-oriented approach to food provisioning which assisted in meeting unique needs and decreasing stigma associated with food insecurity.
- The HIP response illustrated the need for PH to plan with government and community partners to consider food insecurity in future pandemics or emergencies.
- The centrally coordinated approach built upon local knowledge, partnerships, and wider community context was a key to success.

What are the burning research questions for this innovation?

- Did this program reduce the spread of COVID-19 by enabling people to isolate effectively at home or in alternate accommodations?
- How can equity and in particular food security be integrated as key tenets of robust emergency management plans?
- How can multiple levels of government and community agencies work together and define respective roles to reduce reliance on charitable food programs?
- What barriers prevented access to HIP supports and for which populations?
- What prevented a proportion of severely food insecure individuals and households from requiring HIP support? What policy approaches can be used to strengthen these protective factors?

## Data Availability

Not applicable.
